# Ischemic paralysis caused by internal iliac artery embolism after endovascular recanalization for lower extremity arteriosclerosis obliterans: a case report

**DOI:** 10.1186/s42155-026-00783-8

**Published:** 2026-10-13

**Authors:** Jing Wang, Jing Li, Xuemin Zhang, Xiaoming Zhang, Tao Zhang

**Affiliations:** Vascular Surgery Department, Peking University People’s Hospital, Beijing, 100044 China

**Keywords:** Lower extremity arteriosclerosis obliterans, Endovascular recanalization, Internal iliac artery embolism, Ischemic paralysis, Postoperative complication

## Abstract

**Background:**

Lower extremity paralysis is a devastating complication after endovascular therapy for lower extremity arteriosclerosis obliterans (LEAOD). Common mechanisms include ischemia–reperfusion injury, acute arterial embolism, and thrombosis. Paralysis caused by internal iliac artery embolism is exceptionally rare and may be overlooked when postoperative neurologic deficits are initially attributed to spinal or cerebral causes.

**Case presentation:**

An 86-year-old man developed recurrent bilateral intermittent claudication 3 years after endovascular treatment for LEAOD, followed by right toe ulceration. Preoperative computed tomographic angiography showed severe multilevel stenosis and in-stent reocclusion in both lower limbs. He underwent bilateral thrombus aspiration, balloon angioplasty, and catheter-directed thrombolysis. One hour after the procedure, acute left lower limb weakness (muscle strength grade 0–1) and hypoesthesia below the mid-calf occurred. Emergency abdominal-pelvic non-contrast CT, lumbar spine MRI, brain imaging, and lower limb CTA excluded lumbar canal disease and stroke, while suggesting new internal iliac artery occlusion. Emergency thrombus aspiration and balloon angioplasty of the left internal iliac artery restored substantial patency and improved muscle strength to grade III–IV in the thigh and grade III in the leg. Because pain persisted and distal perfusion remained insufficient, femoral endarterectomy and infrapopliteal balloon angioplasty were subsequently performed. After 1 month of rehabilitation, muscle strength remained grade III, with persistent sensory impairment and assisted ambulation.

**Conclusions:**

Internal iliac artery embolism is a rare but clinically important cause of ischemic paralysis after peripheral endovascular intervention. In patients with extensive multilevel occlusive disease, preoperative assessment of internal iliac collateral perfusion and cautious staged procedural planning may reduce risk. Sudden postoperative limb paralysis should prompt urgent spinal and vascular imaging, followed by timely revascularization when ischemia is suspected.

## Background

LEAOD is a common atherosclerotic disease characterized by progressive multisegmental arterial stenosis or occlusion. Advanced or recurrent disease may progress to chronic limb-threatening ischemia (CLTI), with refractory intermittent claudication, rest pain, and non-healing toe ulceration, thereby impairing quality of life and increasing the risk of major amputation [1]. Endovascular recanalization, including balloon angioplasty, stent implantation, atherectomy, and thrombus aspiration, is minimally invasive and can provide favorable short-term patency; consequently, it has become an important treatment option for selected patients with LEAOD [2].

Postoperative lower extremity paralysis after peripheral endovascular intervention is usually attributed to ischemia–reperfusion injury, distal embolization of atherosclerotic material, or acute in situ thrombosis. In patients with external iliac artery occlusion and severe multivessel disease of the superficial femoral, popliteal, anterior tibial, posterior tibial, and peroneal arteries, distal perfusion may depend heavily on collateral flow from the internal iliac artery. Embolization of the internal iliac trunk can therefore produce acute limb ischemia and ischemic lumbosacral plexopathy. Because sudden postoperative paralysis may be mistaken for lumbar radiculopathy or stroke, delayed vascular imaging can compromise salvage. We report a rare case of acute unilateral lower extremity paralysis caused by internal iliac artery thromboembolism after repeat endovascular treatment for extensive multisegmental LEAOD.

## Case presentation

An 86-year-old man presented with bilateral intermittent claudication for 1 month, which progressed to right toe ulceration. Three years earlier, he had undergone left iliac artery balloon angioplasty and stent implantation, as well as left popliteal artery balloon angioplasty and stent implantation. Preoperative lower extremity CTA showed occlusion of the left external iliac and popliteal artery stents, occlusion of both femoral and popliteal arteries and the proximal left profunda femoris artery, and focal severe stenosis or occlusion of both anterior tibial arteries, both peroneal arteries, and the right posterior tibial artery. After multidisciplinary consultation, bilateral lower extremity endovascular recanalization was planned. The requirement for informed consent for participation was waived for this study, and consent for publication was obtained.

**Fig. 1:**
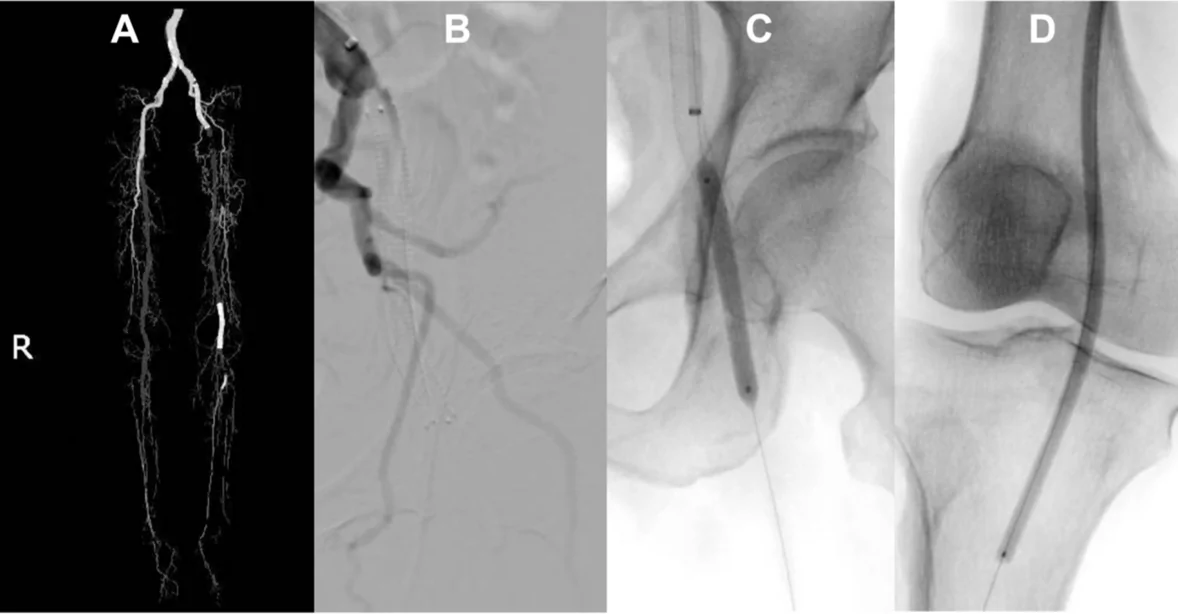
Preoperative computed tomographic angiography of the lower extremities (**A**). Angiography with the vascular sheath tip positioned in the left common iliac artery (**B**). Balloon angioplasty after thromboaspiration of the left external iliac artery (**C**). Balloon angioplasty after thromboaspiration of the right femoropopliteal artery (**D**)

**Fig. 2:**
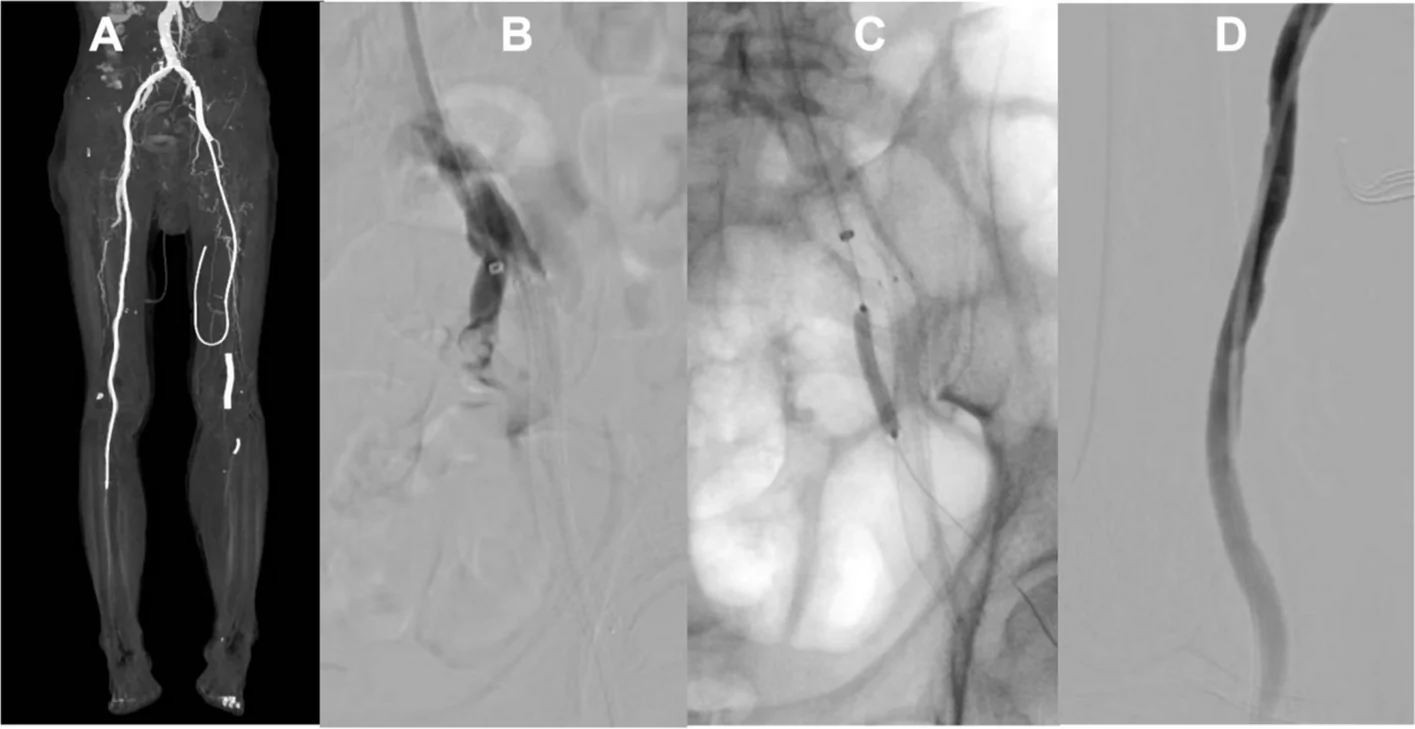
Emergency lower extremity computed tomographic angiography after onset of lower extremity paralysis (**A**). Angiography showed a filling defect in the left internal iliac artery (**B**). Thromboaspiration of the left internal iliac artery followed by balloon angioplasty (**C**). Post-thrombolysis angiography of the right lower extremity arteries showed patent lumens with satisfactory flow velocity (**D**)

With the patient supine, the right femoral artery was punctured retrogradely. Angiography confirmed occlusion of the left external iliac artery stent and distal femoropopliteal arteries. After crossing the occluded external iliac segment, intraluminal thrombus and hyperplastic intima were aspirated with an 8F Rotarex catheter, and residual stenosis was treated with 6-mm and 7-mm balloons. The right femoral puncture site was then closed, and the left femoral artery was punctured to treat the contralateral right femoropopliteal artery; thrombus aspiration, femoropopliteal and infrapopliteal balloon angioplasty, and thrombolysis catheter placement were completed. A dose of 250,000 IU of urokinase was diluted in 20 mL of normal saline and administered as a bolus through the thrombolysis catheter (Fig. 1). One hour postoperatively, sudden left lower limb weakness (quadriceps femoris, hamstring muscles, tibialis anterior, and gastrocnemius grade 0–1) with sensory loss below the mid-calf developed. Physical examination revealed a diminished patellar reflex and an absent Achilles tendon reflex. Further thrombolytic infusion was discontinued immediately. Emergency abdominal and pelvic non-contrast CT, lumbar spine MRI, brain CT/MRI, and lower extremity CTA excluded nerve compression caused by a retroperitoneal or psoas muscle hematoma, stroke, and intraspinal disease but showed new internal iliac artery occlusion (the differential diagnoses are summarized in Table 1). Glucocorticoid therapy was administered during the diagnostic workup. Nine hours postoperatively, emergency thrombus aspiration was performed through a left brachial approach. Angiography showed multiple filling defects and branch occlusions; repeated aspiration with a 6F, 135-cm catheter removed organized thrombus, followed by 2.5 mm × 80 mm balloon angioplasty. Patency of the left internal iliac artery was substantially restored. The total ischemia time was approximately 10 h. Immediately after the procedure, movement improved to grade III–IV in the left thigh and grade III in the left leg, with partial sensory recovery (Figs. 2, 4A, B).

**Table 1:** The differential diagnoses

Diagnosis or mechanism	Case-specific evidence and limitations
Internal iliac occlusion with ischemic plexopathy and/or worsened limb ischemia	New occlusion and immediate partial improvement after reperfusion support an ischemic contribution. Plexus localization and relative contributions remain unconfirmed
Retroperitoneal or psoas/iliacus hematoma with nerve compression	Femoral access and thrombolysis make this relevant. CT was performed, but a negative hematoma finding is stated
Access-site hematoma or pseudoaneurysm	Bilateral femoral punctures were performed, but ultrasonography showed no abnormalities, and there was no significant decrease in hemoglobin levels
Spinal cord ischemia or cerebral ischemia	No explanatory lesion was reported on lumbar MRI and brain CT/MRI
Contrast-associated neurotoxicity	Temporal exposure is compatible but nonspecific; the vascular findings offer an alternative explanation. No targeted assessment is documented; not claimed to be excluded
Position-related femoral or common peroneal compression	Supine positioning is reported. The symptoms did not improve with repositioning, thereby excluding this diagnosis
Compartment syndrome and ischemia–reperfusion injury	Swelling and blisters were reported after hybrid surgery, later than the initial paralysis. The measured delta pressure (diastolic blood pressure minus intracompartmental pressure) was 40 mm Hg, thereby excluding this diagnosis
Progression of in situ thrombosis	The filling defect, together with histopathological confirmation of an organized thrombus in the retrieved material, made acute thrombus formation unlikely
Persistent sciatic artery affecting the ischemic mechanism	Although this represents a relevant anatomical variant, it was ruled out by preoperative CTA

**Fig. 3:**
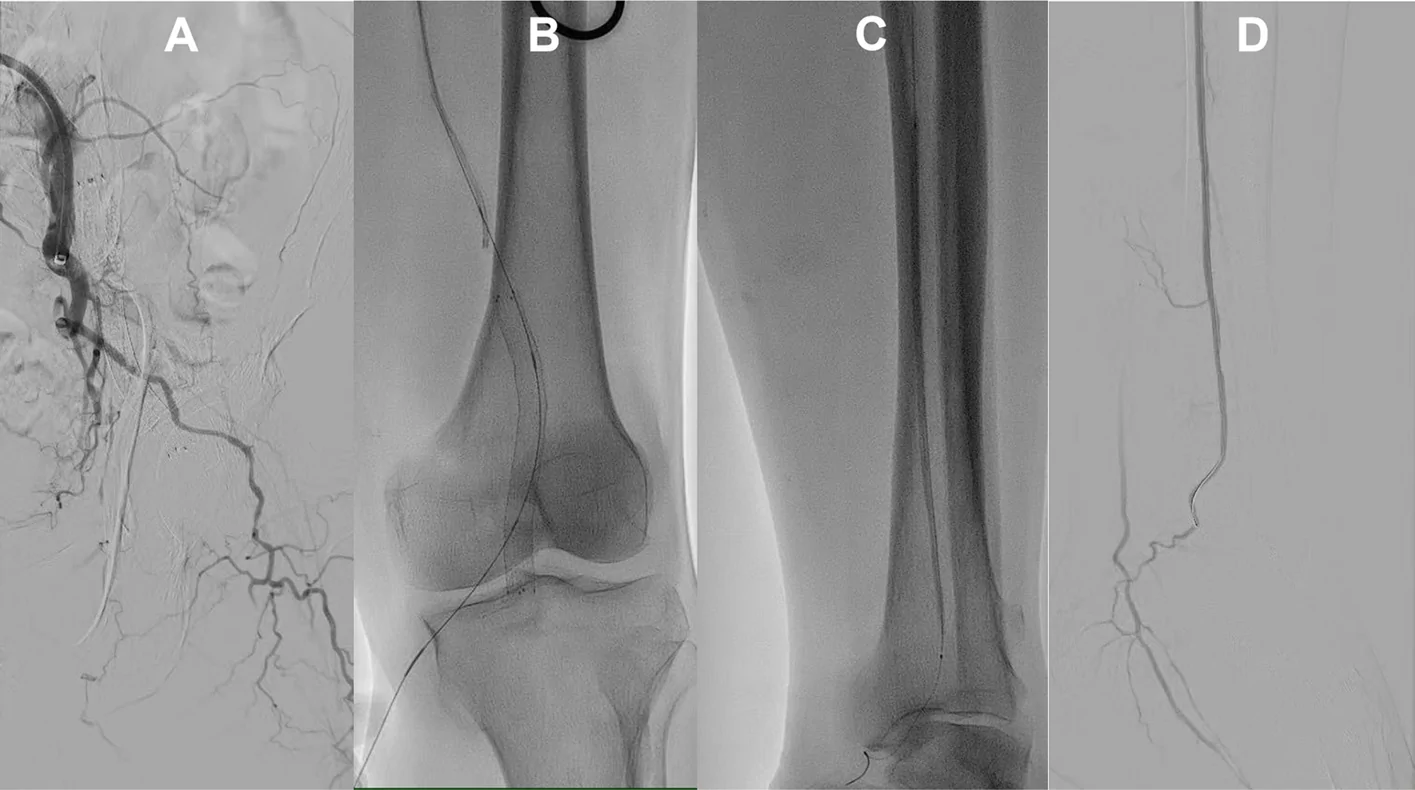
Angiography with the vascular sheath tip positioned in the left common iliac artery (**A**). Retrograde puncture through the left popliteal artery stent; the catheter and guidewire were docked with the antegrade catheter to establish a recanalization route (**B**). Distal recanalization of the peroneal artery using 2-mm and 3-mm balloon catheters (**C**). Post-procedural angiography showed complete opacification of the peroneal artery and partial opacification of the posterior tibial artery (**D**)

**Fig. 4:**
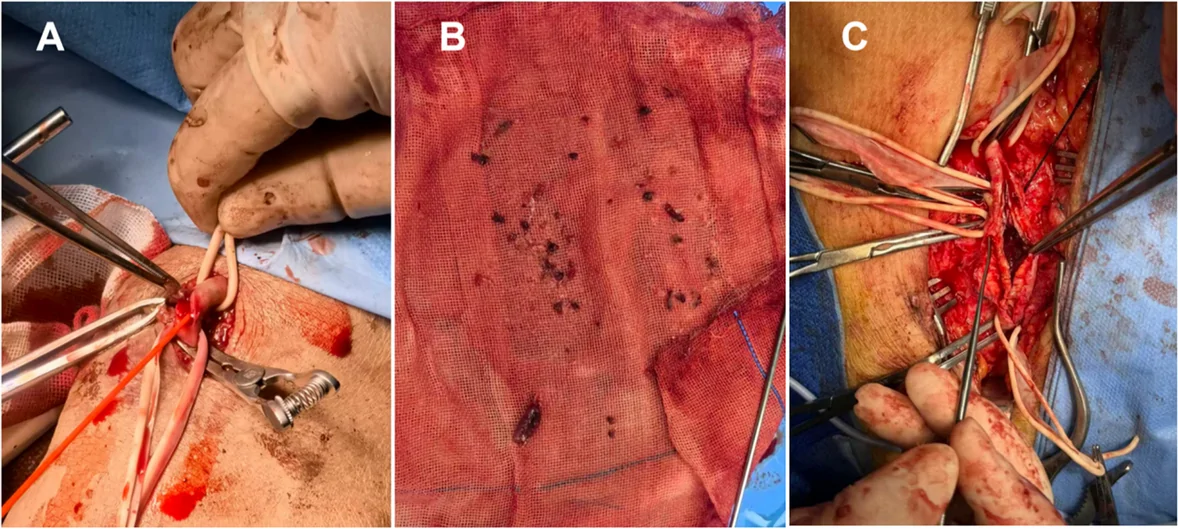
Large chronic thrombus retrieved from the left internal iliac artery through a left brachial artery incision (**A**). Thrombus specimen harvested from the left internal iliac artery (**B**). Endarterectomy of the left femoral artery (**C**)

Although motor function improved, left lower limb pain worsened, response to analgesics was poor, and muscle strength plateaued. Given the severe calcification and complete occlusion of the common femoral artery, successful recanalization with endovascular therapy alone was considered unlikely. Hybrid surgery was therefore performed as an emergency bailout strategy. Angiography again showed a filling defect in the main trunk of the left internal iliac artery, and internal iliac flow alone was considered insufficient for distal perfusion. Left femoral endarterectomy with autologous great saphenous vein patch angioplasty was performed after arteriotomy revealed severe calcification and complete luminal occlusion. Through-and-through access was then established via retrograde puncture of the left popliteal artery stent, and the peroneal and posterior tibial arteries were recanalized distally using 2-mm and 3-mm balloons (Figs. 3, 4C). Postoperatively, swelling and blistering developed in the left lower extremity. The measured delta pressure (diastolic blood pressure minus intracompartmental pressure) was 40 mm Hg, thereby ruling out acute compartment syndrome. After 1 month of rehabilitation, muscle strength remained grade III, tactile, pinprick, and position sensation were diminished, and the patient required assistance with standing, ambulation, and activities of daily living.

## Discussion

Endovascular therapy is widely used for LEAOD, but severe perioperative complications remain clinically important [2]. Neurologic injury associated with internal iliac artery occlusion has been described mainly after endovascular aneurysm repair, in which intentional coverage or embolization of the internal iliac artery may compromise collateral supply to the spinal cord and cause spinal cord infarction with paraplegia [3, 4]. The mechanism in the present case was different. In extensive primary occlusive disease involving the external iliac and multiple distal arteries, limb perfusion may depend primarily on internal iliac collaterals. Embolization of the internal iliac trunk may abruptly interrupt blood supply to the lumbosacral plexus through the iliolumbar and lateral sacral arteries, while simultaneously worsening distal limb ischemia, producing acute motor and sensory loss [5]. Of the two potentially overlapping mechanisms, we considered worsening lower-extremity ischemia due to loss of collateral perfusion from the internal iliac artery to be the predominant mechanism. This is because the lumbosacral plexus is supplied not only by branches of the iliolumbar and lateral sacral arteries but also by the lumbar segmental arteries; consequently, isolated internal iliac artery occlusion rarely results in lumbosacral plexopathy. The sequence of reperfusion followed by rapid neurological recovery strongly supports this mechanism: within minutes after removal of the organized thrombus and restoration of blood flow, muscle strength improved from MRC grade 0–1 to grade 3–4. However, the neurological deficits did not completely resolve, and the subsequent hybrid procedure represents a potential confounding factor; therefore, the final neurological recovery cannot be attributed solely to internal iliac artery revascularization.

Persistent sciatic artery should also be considered in the differential diagnosis. This rare congenital vascular anomaly has a reported prevalence of 0.04–0.06% and results from incomplete regression of the embryonic axial artery. It passes through the greater sciatic foramen, connects the internal iliac artery directly with the popliteal artery, and runs alongside the sciatic nerve [6]. In the complete type, particularly when the superficial femoral or profunda femoris artery is hypoplastic or occluded, acute internal iliac embolization may critically reduce distal limb perfusion and cause sudden motor and sensory deficits. Preoperative CTA ruled out this anatomical variant.

The procedural sequence probably contributed to embolization. After Rotarex thrombectomy of the occluded left external iliac and femoropopliteal arteries through a right common femoral approach, the left femoral artery was used to recanalize the contralateral limb. Multicenter data on Rotarex treatment of in-stent occlusion reported a distal embolization rate of 5.5% [7]. Fragmented thrombus or hyperplastic intima may have been displaced during subsequent catheter and guidewire manipulation across the iliac bifurcation. Retrieval of large organized thrombus fragments during emergency brachial-approach aspiration supports embolization of procedural debris rather than de novo in situ thrombosis within the internal iliac artery.

This case highlights several practical implications for endovascular treatment. In patients with severe bilateral LEAOD and in-stent reocclusion, staged bilateral recanalization should be considered when mechanical thrombectomy is planned. When concomitant treatment of bilateral lesions is planned, prophylactic use of an embolic protection device may be considered. In this case, no such device was used because the possibility of this serious complication was not anticipated by the surgical team. Preoperative CTA should specifically assess the internal iliac ostium, the extent of internal iliac collateral perfusion, and congenital variants such as persistent sciatic artery. If sudden postoperative paralysis occurs, clinicians should not rely solely on neurologic explanations; urgent spinal and whole-limb vascular imaging is required, and revascularization should be performed promptly when arterial occlusion is suspected.

This case report has several limitations. First, the 1-month follow-up period was relatively short for ischemic neurological injury, as neurological function may continue to recover over several months. At the 3-month telephone follow-up, the patient reported no further neurological improvement; however, more detailed, longer-term follow-up data were unavailable. Second, although multiple imaging examinations, including CT, MRI, and CTA, were performed, no direct or indirect physiological evidence, such as perfusion imaging or electrophysiological testing, was available to substantiate the relationship between internal iliac artery occlusion and paralysis. Third, because neither histopathological examination nor compositional analysis was performed, organized thrombus could not be distinguished from atherosclerotic debris or a mixture of both; therefore, attributing the event to procedure-related embolization remains speculative.

## Conclusions

Internal iliac artery embolism is a rare but serious cause of ischemic paralysis after peripheral endovascular intervention. The present case suggests that extensive multilevel occlusive disease can make the lower limb and lumbosacral plexus highly dependent on internal iliac collateral flow. Careful preoperative assessment of internal iliac perfusion, consideration of staged procedures, and meticulous catheter manipulation may reduce embolic risk. Remaining uncertainties include the optimal preventive strategy and timing of revascularization in patients with similar high-risk anatomy; further accumulation of cases is needed.

## Data Availability

All data generated or analyzed during this study are included in this published article.

## Abbreviations

CLTI: Chronic limb-threatening ischemia
CTA: Computed tomographic angiography
EVAR: Endovascular aneurysm repair
LEAOD: Lower extremity arteriosclerosis obliterans
MRI: Magnetic resonance imaging
